# A Critical Overview on Prostaglandin Inhibitors and Their Influence on Pregnancy Results after Insemination and Embryo Transfer in Cows

**DOI:** 10.3390/ani11123368

**Published:** 2021-11-24

**Authors:** Bartłomiej M. Jaśkowski, Adam Opałka, Marek Gehrke, Magdalena Herudzińska, Jarosław Czeladko, Walter Baumgartner, Jędrzej M. Jaśkowski

**Affiliations:** 1 Department of Reproduction and Clinic of Farm Animals, Faculty of Veterinary Medicine, Wroclaw University of Environmental and Life Sciences, 50-366 Wroclaw, Poland; adam.opalka@upwr.edu.pl; 2 Department of Diagnostics and Clinical Sciences, Institute of Veterinary Medicine, Nicolaus Copernicus University, 87-100 Toruń, Poland; gehrke1@o2.pl (M.G.); jedrzej.jaskowski@gmail.com (J.M.J.); 3 Department of Basic and Preclinical Sciences, Institute of Veterinary Medicine, Nicolaus Copernicus University, 87-100 Toruń, Poland; magdalenaherudzinska6@gmail.com; 4 Independent Researcher, Piłsudskiego 26, 16-080 Tykocin, Poland; jaroslaw.czeladko@gmail.com; 5 University Clinic for Ruminants, University of Veterinary Medicine, Veterinaerplatz 1, A-1210 Vienna, Austria; walter.baumgartner@vetmeduni.ac.at

**Keywords:** cattle, NSAIDs, embryo transfer, artificial insemination, pregnancy rate

## Abstract

**Simple Summary:**

Assisted reproductive techniques, such as artificial insemination or embryo transfer have been used in cattle reproduction for decades, but despite many methodological improvements, pregnancy rates have not increased proportionately. One strategy to improve the pregnancy rate after artificial insemination and embryo transfer is to increase the chance of early embryo survival with the use of medications such as nonsteroidal anti-inflammatory drugs. This paper compares the effect of the application of the most frequently used nonsteroidal anti-inflammatory drugs in cattle (flunixin meglumine, carprofen, meloxicam, ibuprofen, aspirin, and sildenafil), as well as of steroid drugs that are used less frequently in cattle reproduction. An evaluation of published reports revealed a range of outcomes that were not always consistent with each other. However, a positive effect of nonsteroidal anti-inflammatory drug treatment on the pregnancy rate in cattle was indicated, especially with the use of flunixin meglumine.

**Abstract:**

Assisted reproductive techniques in cattle, such as artificial insemination (AI) and embryo transfer (ET), are widely used. Despite many years of methodological improvements, the pregnancy rate (PR) in cows has not increased in direct proportion with their development. Among the possibilities to increase the PR is the use of certain steroids and nonsteroidal anti-inflammatory drugs (NSAIDs). The antiluteolytic effect of NSAIDs is achieved by blocking cyclooxygenase, which is involved in the conversion of arachidonic acid to prostaglandins. This article compares the PRs obtained after treatment with the commonly used NSAIDs in cattle, including flunixin meglumine, carprofen, meloxicam, ibuprofen, aspirin, and sildenafil. Studies on the effectiveness of certain steroid drugs on the PR have also been described. The results were not always consistent, and so comparisons between studies were made. In conclusion, flunixin meglumine seems to be an option, and can be recommended for improving ET results, especially in situations of high exposure or susceptibility to stress. Its administration under all circumstances, however, might be pointless and will not lead to the desired effect.

## 1. Introduction

Assisted reproductive techniques, such as artificial insemination (AI) or embryo transfer (ET) in cows, have been used in veterinary practice for many decades. The effectiveness of ET depends on several conditions, including individual, environmental, technical, and technological [1,2]. While the number of embryos transferred is relatively constant worldwide, the results of ET have not improved over the years despite improvements in the welfare of recipients, technical skills, and methods of cryopreservation and embryo production. Furthermore, many ET teams do not achieve satisfactory results. For this reason, numerous attempts have been made to improve the efficiency of ET. One of the more promising solutions is the administration of prostaglandin (PG) inhibitors and their derivatives, mainly steroidal and nonsteroidal anti-inflammatory drugs [3,4,5,6]. Although the first studies on this topic were promising, more cautious opinions have emerged over time. Similarly, nonsteroidal anti-inflammatory drugs are used in attempts to improve the pregnancy rate following AI.

Nonsteroidal anti-inflammatory drugs (NSAIDs) have analgesic, antipyretic and anti-aggregating properties. Steroid anti-inflammatory drugs have similar properties, but due to their numerous side effects in animals [7,8,9,10,11] are less common in use. In addition, due to their anti-inflammatory and immunosuppressive effects, corticosteroids have also been used in animal reproduction and in embryo transfer [12,13,14]. The main action of most NSAIDs is the inhibition of cyclooxygenase (COX), the first in a series of enzymes responsible for the conversion of arachidonic acid (AA) to PGs. Arachidonic acid is converted to prostaglandin G2 (PGG2), prostaglandin H2 (PGH2), and, finally, to prostaglandin F2α (PGF2α) by cyclooxygenase-2 (COX-2) and PGF2α synthase [15]. Among the COX inhibitors, potential candidates that can affect uterine PGF2α release are NSAIDs that act non selectively by competitive inhibition of the COX enzyme via both COX-1 and COX-2 isoforms, i.e., celecoxib, rofecoxib, and valdecoxib, and via preferentially inhibiting COX-2 [5].

Nonsteroidal anti-inflammatory drugs have been demonstrated to delay luteolysis and support embryonic survival [16]. Administration of flunixin meglumine (FM) could impede PGF2α production by the endometrium and potentially improve pregnancy rates in recipient cows by preventing luteolysis. In the era of assisted reproductive techniques being commonly used for farm animal reproduction, there might be additional applications for these drugs in improving the calving results of cows.

This study provides a critical overview of the use of certain PG inhibitors for improving the pregnancy rate in cows after AI and in embryo recipients, with particular emphasis on nonsteroidal anti-inflammatory drugs.

## 2. Nonsteroidal Anti-Inflammatory Drugs

### 2.1. Flunixin Meglumine

Flunixin meglumine (FM) is a nonsteroidal anti-inflammatory drug that inhibits the conversion of AA to PG, preventing its uncontrolled production and, in consequence, embryo death due to progesterone deficiency [6,17]. In beef cows treated with ACTH, it suppresses serum levels of PGF2α metabolites (PGFM) [18]. Similarly, in camels receiving 500 mg of FM intravenously 15 min before ET, the plasma concentration of PGFM remained below 100 pg/mL, while in control recipients it increased to 180 pg/mL 10 min after ET, then decreased to 90–100 pg/mL 110 min after the transfer [19]. Increased luminal concentrations of PGF2α are generally correlated with embryo quality and pregnancy rate. The release of PGF2α from the endometrium is increased after ET [12]. The results of studies carried out on mares indicate that ET causes subclinical inflammation, which can be counteracted by the administration of NSAIDs [20,21]. In studies with sheep, it was found that FM neither prolongs the luteal phase of the estrus cycle nor the lambing rate [22]. Similarly, in cattle, estrus cycle length in FM treated cows was not different from the estrus cycle length in the control group (20 vs. 22 days, respectively) [23]. The most recent studies prove that intramuscular FM treatment in cows had an inhibitory effect on the PGF synthesis within the follicle [24].

Relatively numerous publications show that administration of PGF2α synthase inhibitors prior to ET improves the pregnancy rate in embryo recipient cows [12,13,23,25,26,27]. Scenna et al. [12] reported a pregnancy rate of 65% following FM administration, which was significantly higher than that of the control group (60%). In hot season the PR of Egyptian Baladi cows given i.m. 1.1 mg/kg BW on Day 14 after mating was 60%, while in the control group it was 30% (*p* < 0.05) [23]. Our research, however, showed that administration of FM immediately before ET did not significantly improve the pregnancy rate (*n* = 492; 61.3 vs. 58.5% for FM and control groups, respectively *p* > 0.05) [28]. Furthermore, in larger studies (*n* = 975) in which FM was administered on Days 7 and 16 after insemination, there was also no significant difference in pregnancy rates between the control and treatment groups (60.2% compared to 58.4%, respectively) [29]. Moreover, FM treatment did not improve pregnancy outcomes when there was significantly negative and positive asynchrony between donor and recipient [19]. There was also no difference in PR value (40.2 vs. 44.6% in experimental and control, respectively) for crossbreed bovine embryo recipients, receiving FM immediately after the transfer of in vitro produced grade 1 quality in blastocyst and expanded blastocyst stage embryos [30]. However, the latest analysis shows that NSAIDs at ET were relevant in cows with difficulty in passing the catheter from the cervix during ET and based on the meta-analysis of different NSAIDs treatment results proved that the treatment with NSAIDs was associated on average with 15% more likely PR after ET compared to the control groups [26]. 

Interesting data are provided by Yoon et al. [14], who administered FM or human chorionic gonadotropin (hCG) to recipient heifers and cows prior to ET, with some of the recipients in each group additionally anesthetized with lidocaine. The pregnancy rate (Day 30) in the FM group was comparable to that obtained after hCG administration (76.7% compared to 75.7%, respectively) yet on Day 42, it was higher in the group with FM administration (76.7%) compared to 66.7% in the group that received hCG. The pregnancy rate after administration of FM alone was significantly higher than with FM and lidocaine, both in heifers and cows (72.3 vs. 67.5%, respectively, for heifers and 48.9 vs. 43.6%, respectively, for cows) [14].

Recently, opinions on the effectiveness of FM use have become more nuanced. One study reported that administration of FM increased the pregnancy rate in female recipients, but this was mainly for lower quality embryos as well as embryos at the morula stage, as this effect was not observed with very good-quality embryos or blastocyst-stage embryos [12]. The authors suggest that the poorer quality embryos could have increased susceptibility to the harmful effects of PGF2α. Taking into account that the transfer efficiency of poorer-quality embryos is usually several percent lower than for good-quality embryos, the use of FM in these cases becomes even more important [12]. Other results have been shown by Purcell [31] who found significant FM x location interaction on PR (cows in three different locations treated with FM: Location 1: 89 vs. 57%; Location 2: 69 vs. 64%; Location 3: 64 vs. 67% for FM treatment vs. no treatment, respectively, *p* < 0.01). There was no beneficial effect of FM observed when the preparation was administered on Days 15 and 16 after timed artificial insemination (TAI), and estrus synchronized using the 5 d Co-Synch + CIDR protocol (Day 0—progesterone insert in an unknown phase of the estrus cycle, then intramuscular injection with 1 mg estradiol benzoate (EB), Day 5—injection with 400 IU of equine chorionic gonadotropin (eCG), Day 7—insert removal and intramuscular injection of 1 mg EB, Day 17—insemination) [32,33]. Also, Bülbül et al. [34], reported that giving the recipients 500 mg FM i.m. immediately before the transfer of frozen embryos did not show a significant difference in the pregnancy rate compared to the control recipients receiving saline solution.

The latest data confirm that while administration of FM has a moderately favorable effect on the pregnancy rate in beef cattle, this is only significant in recipient heifers characterized by a violent temperament [35,36]. For example, breeding season PR for excited cows was 88.6%, whereas in calm animals was significantly higher (94.1%) [35]. More recent studies [37,38] have investigated the influence of manipulations accompanying the ET procedure on the pregnancy rate in beef breed recipients characterized by excitable temperament and receiving FM. Recipient cows with a calm temperament had a higher pregnancy rate compared to those with an excited temperament (59.4 vs. 51.7% [37]. The pregnancy rate for excitable cows without FM was lower (46.3%) compared to excitable cows that did receive FM (56.8%), and calm cows that did (59.3%) or did not (59.4%) receive FM [37]. Analysis of a greater group (*n* = 1145) [38] has presented similar observations. Intramuscular and transdermal administration of FM did positively affect the PR of recipients (62.8 vs. 58.7%, respectively) in comparison with the control group (51.2%). Simultaneously, there were no significant differences between routes of administration of FM [38]. The excitable recipients that did not receive FM had increased blood cortisol, substance-P and PGFM levels on Day 7 after ET, and isoprostane 8-epi PGF2a levels during ET and 7 days later. It should be noted that the concentrations of cortisol and substance-P in the blood were positively correlated with the concentration of PGFM, while the concentrations of cortisol, and PGFM in the blood were negatively correlated with progesterone concentration [37]. It is assumed that excitable cows experienced a stress-induced increase in cortisol and substance-P levels, which triggered the release of PGF2a, manifested by an increase in PGFM and suppression of corpus luteum (CL) function, thereby reducing progesterone levels. As a result, this may have caused embryonic losses in some excitable cows that did not receive FM. The females that received FM, regardless of temperament, had significantly lower concentrations of cortisol, substance-P and PGFM, and higher blood progesterone levels [38]. This suggests that FM reduces the effects of stress on the female body, maintains progesterone levels, and, consequently, maintains pregnancy [37]. It cannot be ruled out that the increased concentration of substance P in the bloodstream of excitable cows increased the number of reactive oxygen species and created an unfavorable uterine environment that reduced the likelihood of pregnancy. Administration of FM may inhibit the synthesis of PGF2α produced by the uterine endometrium [39,40] preventing luteolysis and, consequently, delayed heat recovery. However, the percentage of individuals showing estrus after ET did not differ between cows that had received FM and those that were untreated [37].

The studies on FM administration in AI cows have widely varying results. Merrill et al. [6] showed that the transport of beef cows 14 days after AI increased serum cortisol levels, but had no effect on the pregnancy rate. However, in cows treated with FM compared to control cows, the level of PGFM was decreased (39.4 pg/mL vs. 60.6 pg/mL, respectively, *p* < 0.01), and the AI pregnancy rate increased (71 vs. 61%, respectively, *p* < 0.05). Additionally, cows that were transported and previously treated with FM had a higher tendency to improve (*p* = 0.07) pregnancy rate of AI (74%) than those that did not receive FM (66%) [6]. It has been reported that the administration of FM to heifers repeated on Days 15-16 after insemination may be a useful method to improve the pregnancy rate [41], although Thatcher et al. [42] showed that administration of FM had no effect on the PR after TAI [42].

### 2.2. Carprofen

Another nonsteroidal anti-inflammatory agent, carprofen (CAR), was studied solely for the results of calving after insemination. Carprofen is a long effective NSAID with a clinical effect of 12 h. It has a small volume of distribution much longer plasma elimination half-life (30 to 40 h) in cattle than FM and is poorly excreted in milk [43]. Administration of CAR had no significant effect on the concentration of plasma progesterone or on embryo mortality [44], and when administered during the critical period of pregnancy, i.e., on the 14th and 15th Day after insemination, did not have a significant effect on the pregnancy rate in cows [16,45,46]. In cows (>120 days in milk), receiving CAR subcutaneously 14 days after timed AI, the PR (*p* < 0.05) was 42.26% compared to 25.26% in the control group. It is also worth of noting that in high-yield cows (>30 kg of milk per day), the PR was much lower (10.52%). The conclusion was that administration of CAR on the 14th Day after insemination to cows who could not conceive for a long time after parturition may be effective in increasing the rate of conception [46]. In the latest experiment of Abay et al. [47], CAR administration 14 days after AI improved chances (*p* < 0.05) of maintaining the CL in females with body condition score (BCS) ≤ 2.5 (81.7 vs. 70.7% respectively for CAR and control group), whereas for animals with BCS > 2.5 CAR treatment decreased CL maintaining chances compared to the control (74.5 vs. 90.5%, respectively, *p* < 0.05). However, a positive effect of CAR has been found by the number of services on pregnancy losses. Pregnancy losses 28–32 and 55–60 days after insemination were higher in cows inseminated thrice or four and more times in the control group (11.8% and 16.7%, respectively) compared to those treated with CAR (0% and 4.8%, respectively) [47]. Conversely, subcutaneous administration of CAR (1.4 mg/kg body weight (BW)) did not have a positive effect on the results of calving in cows, while intrauterine administration with a special catheter had a negative effect on the results of the first insemination [16,48]. Carprofen administered 12 h after delivery had a positive effect on a higher PR after the first insemination (35 vs. 10%, respectively, *p* < 0.001) [49].

### 2.3. Meloxicam

Meloxicam (MEL), a cyclooxygenase inhibitor with a relatively long half-life, can be used in place of FM in clinical practice [26]. In Nelore heifers (*Bos indicus*)*,* in which ET is more difficult than in *Bos taurus* cattle heifers due to the morphological features of the cervix, and therefore requires longer manipulation, administration of MEL should improve the pregnancy rate by lowering the PGF2α level. Meloxicam was administered one hour before transfer (200 mg per head), and the serum concentration of 13,14-dihydro-15-keto-PGF2α (PGFM) was assessed immediately before the transfer, and then 4 and 8 h after the transfer. Administration of MEL resulted in a significant increase in the pregnancy rate on Day 30 after estrus in heifers (72.1 vs. 45.2%, respectively for MEL and control groups). In animals with a short time of the cervical pass, administration of MEL resulted in much higher PR (90.48%) than in the control group (47.62%) (*p* < 0.01). No differences were found in grade II cervices (54.54 vs. 42.86%, respectively, for MEL and control groups) [5]. Similar results were noted by Aguiar et al. [3]. Although administration of MEL did not influence PR in animals with easy cervices, in general, animals treated with MEL had higher PR (66.7 vs. 49%, respectively, for MEL and control, *p* < 0.01). Even clearer differences were noted when there was a difficult passage of the gun through the cervix (>80 s). The PR for difficult passage animals treated with MEL was 78.84% compared to 21.15% for the control group [3]. In contrast to FM, administration of MEL to cows on Day 15 after insemination caused a dramatic decrease in the pregnancy rate from 52 to 24.3%, with the inhibitory effect of MEL lasting for up to 72 h [50]. However, cows with mastitis treated with MEL had a higher chance of conceiving to first AI (33 vs. 24%, for MEL and control groups, respectively, *p* < 0.05) and had a higher PR at Day 120 (42 vs. 33%, for MEL and control groups, respectively, *p* = 0.05) [51].

### 2.4. Ibuprofen, Aspirin, Sildenafil, Tolfenamic Acid

In women preparing for ET, the ultrasound assessment of endometrial thickness has some practical significance, as an endometrium that is too thin or too thick in the late proliferative phase may have a negative effect on ET results. In consequence, low-dose aspirin, sildenafil, and vaginal administration of estradiol has been used in attempts to increase the endometrial thickness in women [52]. Previous data showed that women undergoing IVF and receiving a daily oral dose of 75 or 100 mg aspirin had a higher pregnancy rate than women receiving placebo [53,54]. Likewise, in cows receiving aspirin orally at 50 mg/kg BW on Days 14 and 15 after natural mating, the percentage of pregnant cows was 40% compared to 30% in the control group (*p* < 0.05). At the same time, the length of the estrus cycle did not differ significantly compared to the control group [23]. Similarly, in animals, a significant improvement in pregnancy results was obtained in embryo recipient heifers that received 5 mg ibuprofen intramuscularly [55]. These results are consistent with other studies, in which the administration of strong COX-1 and/or COX-2 inhibitors, such as aspirin or ibuprofen, to heifers and cows during ET improved the pregnancy rate [56,57]. However, the results of another study [58] do not confirm the positive influence of aspirin administration on the pregnancy rate in lactating dairy cows experiencing heat stress.

Tolfenamic acid (TA) is an NSAID belonging to the group of fenamates. It is used during inflammations associated mainly with respiratory disease [59,60]. The effect of TA and FM in PR and embryo survival of recipient mice subjected to ET was evaluated. The use of TA at the time of ET improved both PR and the number of live pups in recipient mice, with optimal effects observed with FM. The authors suggested that the use of TA had beneficial effects on the maintenance of pregnancy and embryo survival in recipient mice, which should be taken into account for further studies in other mammalian females [61].

More detailed data on the efficacy of AI and ET in cows in combination with the applied NSAIDs are presented in Table 1.

## 3. Steroids

Glucocorticoids have been used extensively in veterinary practice since the middle of the 20th century [62]. They have offered the practitioners of veterinary medicine a useful tool for reducing pathological changes seen in infectious and metabolic diseases [62]. They regulate many of the processes required for successful embryo implantation, as well as for the subsequent growth and development of the fetus and placenta [63]. In utero, the endometrium, placenta, and embryo/fetus are each exposed to physiological glucocorticoids arising from either maternal or fetal adrenal glands. It has been shown that glucocorticoids regulate the synthesis of prostaglandins that have been implicated to play critical roles during implantation by increasing stromal vascular permeability and in the initiation of parturition. In the first trimester in human cytotrophoblasts, cortisol can suppress the synthesis of pro-inflammatory interleukin (IL)-1b [64,65]. It is now known that most glucocorticoids given at high levels can act as abortifacients [66]. The potent glucocorticoids dexamethasone (DEX) and flumethasone appear to be among the most active parturition inducing agents [62,67].

Steroids are less commonly used than NSAIDs as agents to influence pregnancy rates after ET. Prednisolone, however, was applied in studies on women embryo recipients [68], while in cattle reproduction, a positive effect of the addition of DEX to embryo straws on the pregnancy rate in the embryo recipients has been described [69].

Prednisolone is a glucocorticoid produced in the zona fasciculata of the adrenal cortex under the influence of adrenocorticotropic hormone (ACTH), which regulates the metabolism of proteins, carbohydrates, and fats. While it has not been applied in recipient cows, there are reports of its use in woman embryo recipients, but the addition of low-dose prednisolone treatment before and after ET did not appear to have a significant effect on the pregnancy rate [70].

The original method of improving the results of ET was used by Roh et al. [69]. Day 5 cattle embryos were cultured to Day 10 in Christopher Rozenkrans 2aa (CR2aa) medium (control group) supplemented with 100 nM dexamethasone and/or 1000 U/mL recombinant human leukemia inhibitory factor (rhLIF). To determine the effect of PGF2α, 100 ng/mL PGF2α was added to the medium of the experimental groups. Regardless of the experimental group and the addition of PG, there was a significantly higher percentage of blastocysts at the end of the observation phase on the 7th Day of embryonic development compared to the control group. However, the addition of PGF2α did have a negative effect, resulting in fewer than half the number of hatched blastocysts than in the control group on the 10th Day of the study. The adverse effect of PG was counteracted by the re-addition of DEX, rhLIF, or DEX + rhLIF. In the second part of the experiment, the effect of intrauterine administration of DEX and rhLIF on the peripheral blood concentration of PGF2a was determined. Dexamethasone and rhLIF were added to the medium in the transfer straws and PGF2α concentration was determined twice, 60 min before and after ET. The control group had significantly higher levels of PGF2α than females from the DEX and rhLIF group. At the same time, the DEX + rhLIF group had an increased pregnancy rate compared to the control animals. These studies indicate that the intrauterine introduction of dexamethasone or rhLIF may prevent an increase in PGF2a concentration in the peripheral blood, positively influencing the pregnancy rate. In another examination, DEX supplementation (its three concentrations—0.01, 0.1 and 1 mg/mL) was initially evaluated for their effect in IVC of bovine embryos [71]. Dexamethasone did not reduce apoptosis rates, however, it had a positive impact on development kinetics both on Days 4 and 7 of embryo culture. Culture in the presence of 0.1 μg/mL DEX yielded the highest percent (53.9%; *p* < 0.05) of good quality embryos. Notably, embryo culture in the presence of DEX did result in expanded and hatched blastocysts presenting higher cell numbers (*p* ≤ 0.05), consistent with a positive effect on development. Presumably, such an effect resulted from increased cell proliferation rather than direct inhibition of apoptosis. Further studies can evaluate the mechanisms by which glucocorticoids may affect embryo development as DEX supplementation could become a tool to improve in vitro embryo yield in mammalian species.

## 4. Conclusions

Regardless of the drug used, any possible positive effects may be counteracted by the influence of some individual and environmental factors. The use of NSAIDs immediately prior to ET and AI may have a positive effect on the pregnancy rate of cows, with the favorable influence of FM on pregnancy being the relatively best documented. It is worth noting that FM increases the effectiveness of transfer, especially in the case of poorer quality embryos, and thus, it can be recommended for improving ET results, especially in situations of high exposure or susceptibility to stress. Its general administration, however, may not lead to the desired effect. The results of ET after administration of other NSAIDs also seem promising, but the data are insufficient and need more research. Despite their positive effect on early embryos, steroids seem to be of less interest for their use during ET. Furthermore, in the case of those ET teams that achieve above-average results, interventions with steroids and NSAIDs to improve the pregnancy rate may not produce the expected positive effect. The beneficial effects of NSAIDs on the results of AI are less documented. It seems that they can find application in relation to selected groups of animals, e.g., repeating cows, or those exposed to increased pressure of environmental factors, yet their use in artificially inseminated animals without reproducing problems is pointless.

## Figures and Tables

**Table 1:** Comparison of pregnancy results after administration of different NSAIDs.

Number and Cathegory of Animals	Treatment Schedule	Preparation	Dosage and Route of Administration	PR—Experiment:Control (References)
Holstein heifers (*n* = 391)	14/15–15/16 days after AI	FM	2.2 mg FM i.m./kg BW	54.8:58.2% (*p* = 0.5) [Krueger & Hawieser 2010]
Angus crossbreeds (*n* = 1145)	Direct before ET	FM	1.1 mg/kg BW i.m.	62.8:51.2% (*p* = 0.01)
	Direct before ET	FM	3.3 mg/kg BW transdermal	58.7:51.2% (*p* = 0.04) [Kasimanickam et al. 2019]
Brown Swiss heifers (*n* = 39)	5 min before ET	FM	500 mg FM i.m.	50.0:52.6% (*p* > 0.05) [Bülbül at al. 2010]
Angus crossbreed cows (*n* = 483)	4–6h before transport and 14 days after AI	FM	1.1 mg/kg BW i.m.	71:61% (*p* < 0.05) [Merrill et al. 2007]
Angus heifers (*n* = 1,221)	13 days after AI	FM	1.1 mg/kg BW i.m.	66:72% (*p* = 0.02) [Geary et al. 2010]
Angus-cross cows (*n* = 705)	13 days after AI	FM	1.1 mg/kg BW i.m.	57:58% (*p* = 0.8) [Geary et al. 2010]
Holstein heifers (*n* = 52)	15/16 days after AI	FM	2x 1.1 mg/kg BW i.m.	76.9:50.0% (*p* < 0.05) [Guzeloglu et al. 2007]
Crossbreed cows (*n* = 184)	Direct after ET	FM	1.1 mg/kg BW i.m.	40.2:44.6% (*p* > 0.05) [Cardoso et al. 2020]
Egyptian Baladi cows (*n* = 20)	14 days after mating	FM	1.1 mg/kg BW i.m.	60:30% (*p* < 0.05) [Damarany & Ghanen 2020]
Nelore x Caracu heifers (*n* = 207)	Direct after ET	MEL	200 mg MEL i.m.	66.7:49.0% (*p* < 0.01) [Aguiar et al. 2013]
Holstein heifers (*n* = 85)	Direct after AI	MEL	0.5 mg/kg BW s.c.	24.3:52.0% (*p* < 0.01) [Erdem & Guzeloglu 2010]
Nelore heifers (*n* = 85)	One hour before ET	MEL	200 mg of MEL i.m.	90.48:42.86%—Grade 1 recipients only (*p* < 0.01)
	One hour before ET	MEL	200 mg of MEL i.m.	54.54:42.86%—Grade II recipients only (*p* < 0.01) [Lopes et al. 2015]
Holstein cows and heifers (*n* = 970)	Direct after AI	CAR	1.4 mg/kg BW s.c.	42.2:45.1% (*p* > 0.05)
	12-24 h post-AI	CAR	1.4 mg/kg BW intra uterine	38.3:45.1% (*p* = 0.08) [Heuwieser at al. 2011]
Holstein cows (*n* = 556)	14/15 days post AI (hot season)	Aspirin	187.2 g per cow, p.o.	21.7:27.5% (*p* > 0.05) [Spencer et al. 2020]
Egyptian Baladi cows (*n* = 20)	14/15 days after mating	Aspirin	50 mg/kg BW orally	40:30% (*p* < 0.05) [Damarany & Ghanen 2020]
Nelore cows (*n* = 75)	One hour before ET	Ibuprofen	5 mg/kg BW i.m.	43.3:16.0% (*p* < 0.05)
		Ibuprofen	Array polymeric release of controlled ibuprofen, s.c.	14.2:16.0% (*p* > 0.05) [Navarez et al. 2010]

PR—pregnancy rate, FM—Flunixin meglumine, MEL—meloxicam, CAR—carprofen, BW—body weight.
